# Dynamic Adsorption of a Cationic Dye onto Wool Fibers as Column-Filling Media: Response Surface Optimization and Fixed-Bed Adsorption Modeling

**DOI:** 10.3390/ma17040790

**Published:** 2024-02-06

**Authors:** Bogdan-Constantin Condurache, Corneliu Cojocaru, Alexandra Bargan, Petrisor Samoila, Valeria Harabagiu

**Affiliations:** “Petru Poni” Institute of Macromolecular Chemistry, 41A Grigore Ghica Voda Alley, 700487 Iasi, Romania; condurache.bogdan@icmpp.ro (B.-C.C.); anistor@icmpp.ro (A.B.); samoila.petrisor@icmpp.ro (P.S.); hvaleria@icmpp.ro (V.H.)

**Keywords:** wool fibers, adsorption, kinetics, isotherms, thermodynamics, fixed-bed column, design of experiments, model-based optimization

## Abstract

This study reports a simple and low-cost method for water purification using recyclable natural fibers (coarse wool fibers) as column-filling media for adsorption in the dynamic mode. As an instance of a dissolved organic pollutant, a cationic dye (basic blue 9, BB9) was assayed. According to the Langmuir isotherm (recorded at 300 K), the calculated maximum adsorption capacity of the fibrous material was found to be 24.86 mg/g for the retention of BB9. Response surface methodology (RSM) was employed for the design of experiments and the model-based optimization of the adsorption process performed in the dynamic regime (fixed-bed column). The optimal conditions provided by RSM indicated an adsorbent column height of *H* = 13.5 cm and a feed flow rate of *F*_v_ = 3 mL/min; these operating parameters ensured a color removal efficiency of 92.56% after 240 min of contact time. The recorded breakthrough curve under the optimal conditions was further interpolated using five quantitative mathematical models (Adams–Bohart, Thomas, Yoon–Nelson, Yan, and Clark) to assess the dynamic behaviors in the fixed-bed column. The best goodness-of-fit was achieved for the Thomas and Yoon–Nelson models. Thus, the coarse wool fibers used in a fixed bed demonstrated a relevant efficiency in the removal of cationic organic pollutants from contaminated water.

## 1. Introduction

The raw wool fibers of coarse grades (with a diameter > 31 μm), though abundant in nature, often find themselves sidelined in the textile industry due to their inherent limitations. In many cases, the coarse wool fibers represent problematic wastes that are often burned or randomly dumped [1]. On the other hand, wool fibers (WF) possess many distinct attributes [2], such as natural origin, cheapness, keratin-based composition, cuticle-like texture, biodegradability, renewability, non-toxic nature, good mechanical features, and recyclability. It turns out that this overlooked byproduct holds untapped potential for sustainable practices and environmental solutions. Hence, harnessing raw wool fibers as a valuable product for environmental applications is of practical importance.

In the last several decades, the utilization of wool-based materials as low-cost adsorbents for wastewater treatment has attracted attention. To this end, wool fibers of different proveniences were employed successfully in combating accidental pollution as oil spill sorbents [3,4,5,6,7]. These studies also pointed out the possibility of recycling the wool-based sorbents after recovering retained oils from spent materials through centrifugation [3] or squeezing [5,6,7] methods. Likewise, the pristine or chemically modified wool fibers demonstrated their applicability as useable adsorbents for the removal of heavy metal ions [8,9,10,11] and dyes [11,12,13,14] from wastewater.

For instance, in a previous study [11], Alizarin red S (ARS) dye was removed from wastewater with an efficiency of 93.2% when using sheep wool as the sorbent material. Moreover, the ARS-loaded wool was then employed as an adsorbent for the removal of Cr(VI) ions from industrial effluent (i.e., secondary adsorption) [11]. Podjava and co-workers [12] reported the efficient removing of Congo Red (CR) dye from wastewater using Latvian sheep wool fiber as a cheap natural adsorbent. Moreover, these authors also suggested the mechanism of intermolecular interactions between CR molecules and keratine, which was based on electrostatic interactions, hydrophobic contacts, π–π stacking, and hydrogen bonding [12]. In another study, Wen and collaborators [13] investigated the adsorption behavior of wool powders for three acid dyes (C. I. Acid Red 88, C. I. Acid Red 13, and C. I. Acid Red 18), as well as a basic dye (methylene blue). It was found that the maximum adsorption of acid dyes and methylene blue by wool powders occurred at pH levels of 2.5 and pH 7.5, respectively. In addition, the dyes–absorption equilibrium models were in good agreement with the Langmuir isotherm. The adsorption performance of fine wool powders was compared with the performance of activated charcoal, and it was found to be satisfactory [13]. The previous study [14] showed the applicability of wool fibers for the adsorption of some azo dyes (acid violet 17, acid blue 90, acid red 1, and direct red 80) from aqueous solutions, depending on shaking time, dye concentration, and temperature. These outcomes evidenced that the wool fiber possessed a heterogeneous surface with adsorption sites of different activity [14]. The majority of the applications of wool fibers as adsorbents for organic ionic dye removal from wastewater were carried out in batch modes. Little attention was paid to the wool as the fixed bed in dynamic adsorption (i.e., adsorption in the column). Generally, the adsorbent–adsorbate interaction can be explored through batch adsorption assays or via dynamic fixed-bed experiments [15]. Typically, batch tests are performed to assess the effectiveness of a particular adsorbent in removing a target pollutant, as well as to determine the maximum adsorption capacity. On the other hand, the adsorption in the dynamic regime for the fixed-bed systems is conducted under continuous flow conditions, which are of more interest for industrial applications. The performance of the fixed-bed column can be described via the dynamic adsorption profile that represents the effluent concentration against time. This is known as the breakthrough curve (BTC), and it is an important feature for establishing the operating conditions of fixed-bed columns [15].

This study aimed to explore coarse wool fibers as a low-cost adsorbent for the removal of cationic dye from wastewater, placing a special focus on fixed-bed adsorption. Optimal operating conditions for the fixed-bed column were established using the response surface methodology. The optimal BTC profile was modeled using various quantitative mathematical models in order to get insights and enhance the fixed-bed system.

## 2. Materials and Methods

### 2.1. Materials

Raw wool fibers of very coarse grades (with a maximum fiber diameter greater than 36.2 μm) from a regional sheep farmer (Todireşti, Vaslui, Romania) were scoured through a common cleaning process. This included the manual removal of macroscopic solid impurities, followed by the washing of the raw wool several times with warm distilled water (45 ± 2 °C). Ultimately, the scoured wool fibers were dried in the laboratory oven at 35 °C for 24 h and were then stored in a desiccator for further use.

The cationic dye chosen as the target adsorbate was basic blue 9 (BB9), also called methylene blue, with the molecular formula C_16_H_18_ClN_3_S and a molecular weight of 319.9 g/mol, supplied by Sigma-Aldrich/Merck, St. Louis, MO, USA (CAS 7220-79-3). A stock solution of BB9 dye was obtained at a concentration of 1000 mg/L, from which the working aqueous solutions were prepared via dilution at the required concentrations. The common chemical reagents of an analytical grade (NaOH, NaCl, HCl, C_6_H_8_O_7_, CH_3_OH—acquired from Sigma-Aldrich/Merck) were used as received, without any further purification.

### 2.2. Characterization Methods

Raw wool fibers were characterized from morphological and structural standpoints. The morphology and solid surface of fibers were examined by scanning electron microscopy (SEM) using an ESCM Quanta 200 spectrometer, coupled with an energy-dispersive X-ray (EDX) module (FEI Company, Brno, Czech Republic). The micrographs provided by SEM were further analyzed using the ImageJ (version 1.45s) open-source software to build the histogram of fiber diameter distribution. The Fourier transform infrared spectrum (FTIR) of the wool fiber was registered in the mid-infrared region (4000–600 cm^−1^) using a Bruker Vertex 70 FTIR spectrometer (Bruker Corporation, Ettlingen, Germany). Dynamic vapor sorption (DVS) analysis was employed to inspect the behavior of wool fibers in the presence of moisture. In this respect, the adsorption capacity of wool fibers for the water vapors, in a dynamic regime, was determined using the fully automated gravimetric equipment IGAsorp, made by Hiden Analytical (Warrington, UK). The mechanical strength of the wool fibers was tested using the Instron 3365 equipment (Instron Company, Norwood, MA, USA). Additionally, optical microscopy was employed to inspect some visual changes in fiber surfaces during adsorption. To this end, the digital optical microscope (Conrad USB, Wels, Austria) and the polarized light optical microscope (Leica Microsystems, Wetzlar, Germany) were used. In the course of the adsorption experiments, the concentrations of the BB9 dye were evaluated by recording the absorbance on a UV-Vis spectrophotometer Hitachi UV-2910 (Hitachi High Technologies Company, Tokyo, Japan) at a 664 nm wavelength.

### 2.3. Adsorption Experiments in Batch Mode

Batch adsorption tests were first performed to ascertain the effectiveness of coarse wool fibers when removing the target organic pollutant (BB9), and to assess the kinetics, equilibrium, and thermodynamics of the adsorption. To this end, an orbital shaker BIOSAN ES-20/60 (Biosan Company, Riga, Latvia), equipped with a temperature-control system, was employed to stir (at 150 rpm) the working solutions (50 mL) that contained the adsorbent (wool) and the adsorbate (BB9). At the end of the adsorption assay, the spent adsorbent (wool) was removed from the liquid phase, and the purified solution was analyzed for the leftover concentration of the BB9 dye. The adsorption capacity *q* (mg/g) of the coarse wool fibers for the uptake of BB9 was determined as

(1) $$q=\frac{(C_{0}-C)\times V}{m\times 1000}$$

where *C*_0_ and *C* (mg/L) denote the dye concentrations in the initial and final solutions, respectively; *V* (mL) is the volume of the aqueous solution; and *m* (g) designates the mass of the adsorbent (wool sample).

The batch-mode adsorption kinetics were carried out for 50 mg/L of the initial concentration of the pollutant (BB9) and an adsorbent dosage of 0.5% *w*/*v*. For the adsorption isotherms, the experiments were performed considering various initial BB9 concentrations, ranging from 25 mg/L to 700 mg/L, whereas the adsorbent dose was fixed at 0.2% *w*/*v*.

Batch adsorption experiments were also performed to assess the thermodynamic parameters (Gibbs free energy Δ*G_ad_*, enthalpy Δ*H_ad_*, and entropy Δ*S_ad_*) of the process. Hence, the change in the Gibbs free energy of adsorption (Δ*G_ad_*) was ascertained as [16,17]

(2) $${\Delta G}_{ad}=-R_{g}T\;ln(K_{ML})$$

where *T* denotes the absolute temperature (K), *R_g_* is the universal gas constant (*R_g_* = 8.314 J/(K∙mol)), and *K_ML_* is the modified Langmuir constant according to the revised isotherm [16]. Assuming the Va not Hoff isochore equation, the change in the enthalpy of adsorption (Δ*H_ad_*) can be assessed as follows [17]:

(3) $${\Delta H}_{ad}=\frac{{-R}_{g}ln\left(\frac{K_{ML,{\;T}_{2}}}{K_{ML,{\;T}_{1}}}\right)}{\frac{1}{T_{2}}-\frac{1}{T_{1}}}\;$$

where *T*_1_ and *T*_2_ designate two distinct levels of temperature; consequently, $K_{ML,{\;T}_{1}}$ and $K_{ML,{\;T}_{2}}$ denote the equilibrium constants ascertained for those two temperatures.

Ultimately, the variation of the adsorption entropy (Δ*S_ad_*) was calculated from the basic thermodynamic equation as [17]

(4) $${\Delta S}_{ad}=\frac{\Delta H_{ad}-\Delta G_{ad}}{T}\;$$

### 2.4. Adsorption Experiments in Dynamic Mode (Fixed-Bed Column)

The fixed-bed experiments were carried out by employing a glass column with an inner diameter of 1.2 cm and a total length of 20 cm. The fixed bed assembled from wool fibers was inserted into the glass column as the adsorbent material. These fibers of coarse grades were packed into the column with a packing density of 0.126 g/cm^3^. To point out the dynamic adsorption profiles (BTC curves), the relative concentration (the ratio between BB9 concentrations in effluent and influent, *C*/*C*_0_) was represented against the contact time (*t*).

## 3. Results and Discussion

### 3.1. Characteristics of the Adsorbent (Wool Fibers)

**Figure 1:**
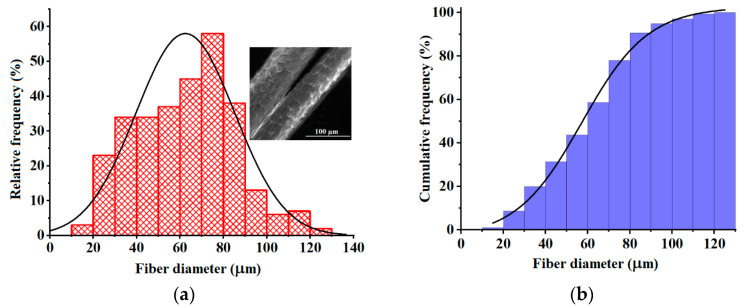
Histograms of fiber diameter distributions: (**a**) Relative frequency histogram; (**b**) Cumulative frequency histogram. The inset image represents the SEM micrograph of coarse wool fibers.

Figure 1 displays the histograms of fiber diameter distribution (Figure 1a,b), as well as an example of an SEM micrograph of wool fibers given as the inset. These histograms were built by counting 300 fiber diameters from different SEM images of wool samples. The inset image in Figure 1a shows cuticle-like structures on the top surfaces, which are specific to the wool fibers [2]. As given in the histogram from Figure 1a, the wool fiber diameters varied from 19 μm to 128 μm, following a normal distribution. The histogram analysis disclosed an average fiber diameter equal to 63 μm. The cumulative histogram (Figure 1b) indicated that 50% of the inspecting fibers bestowed diameters greater than 57 μm. According to the classification mentioned by Rajabinejad et al. [1], wool of a very coarse grade has a maximum fiber diameter of over 36.2 μm. Hence, the wool explored in this study is of a very coarse grade, as evidenced by the histogram analysis (Figure 1).

**Figure 2:**
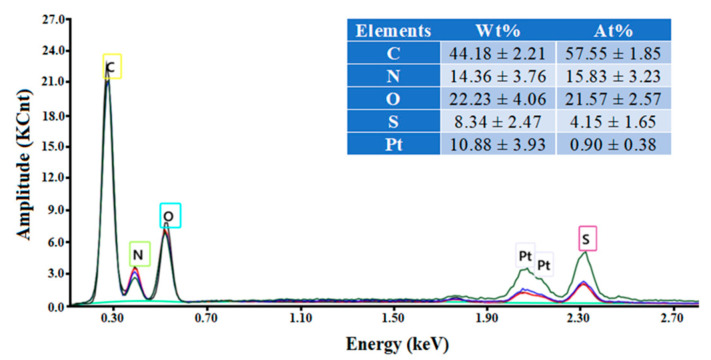
EDX spectrum and the elemental composition (inset) of the coarse wool fiber.

The EDX spectrum of the wool fiber (Figure 2) disclosed the presence of all expected chemical elements, i.e., C, O, N, S (Pt is due to fiber metallization). The EDX characterization technique provides the qualitative elemental analysis, which is surface specific (i.e., the estimation has a local character). The weight and atomic percentages reported in the inset table (in Figure 2) represent the locally averaged values. These are in reasonable agreement with the general chemical composition of wool reported in the literature [2].

**Figure 3:**
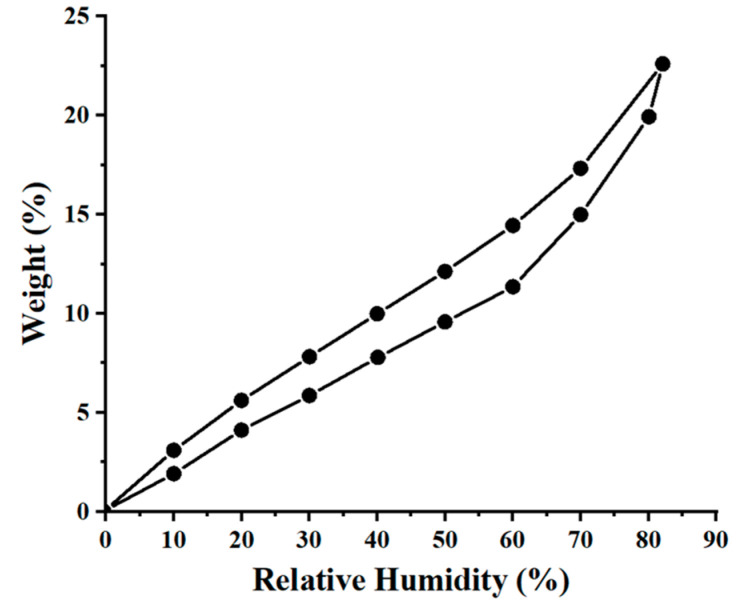
Adsorption and desorption isotherms of water vapors onto the wool fibers (T = 298 K).

Dynamic vapors sorption (DVS) measurements for the wool sample, in the relative humidity (RH) interval of 0 to 90%, are reported in Figure 3, as the adsorption and desorption branches of the water vapors. The special structure of wool fibers (the exterior of the fiber is hydrophobic, while the interior of the fiber is hygroscopic) determines its behavior in the presence of moisture. The DVS measurements were completed in order to assess the specific surface area of the wool fibers explored in this study. Next, the BET method (Brunauer–Emmett–Teller) was employed to estimate the surface area, based on the water vapor desorption in the dynamic conditions, and for the relative humidity range of 10–40%. Thus, the DVS measurements and BET method indicated a specific surface area for wool fibers equal to 275 m^2^/g.

The mechanical tests on coarse wool fibers revealed an elongation at breaks (or tensile strain) of 28.7% and 39.9% for dry wool fiber (RH = 0%) and wet wool fiber (RH = 55%), respectively. Hence, the wool fiber can be stretched more in conditions of greater relative humidity (RH). More details regarding the mechanical characterization are reported in the Supplementary Materials (SM) in Figure S1 and Table S1.

In addition, Fourier transform infrared spectroscopy (FTIR) was employed to gain the structural information on the investigated coarse wool fiber. The infrared (IR) spectrum of the coarse wool fiber is reported in Figure S2 (Supplementary Materials). The identified main absorption bands, characteristic of the wool fiber, are mentioned next. These broadband signals correspond to various molecular vibrations: O–H stretching vibrations (centered at 3438 cm^−1^), which overlap the absorption bands of amide A (3252 cm^−1^) and B (3077 cm^−1^); C–H stretching vibrations (three bands at 2962, 2922 and 2853 cm^−1^); a shoulder at 1748 cm^−1^, characteristic of carboxylic groups (–COO^−^) that are specific to aspartic and glutamic amino acid residues form keratin; the C=O band associated with amide I (1647 cm^−1^); the C-NH band of amide II (1539 cm^−1^); two bands (1448 and 1386 cm^−1^) attributed to protonated groups –NH_3_^+^, emerging from the ionic bonds established between the structural units of the substituted peptides with carboxylic groups of aspartic acid and amino groups of lysine; and the band specific to the vibrations of amide III (1262 cm^−1^). Likewise, other vibrational bands were identified: broad bands centered at 1161, 1117, and 1040 cm^−1^ (corresponding to the cysteine groups indicating the existence of oxidized cystine in raw wool) [18]; bands at 900, 840, 768, and 700 cm^−1^, corresponding to out-of-plane C–H bending [19]; the last relevant band 620 cm^−1^, corresponding to the C–S vibrations; and the low-intensity bands at 525 cm^−1^ and 462 cm^−1^, attributed to S–S stretching vibrations [19]. Additional details regarding the attributions of IR peaks for wool-based materials may be found elsewhere [10].

### 3.2. Adsorption in Batch Mode

Batch-mode preliminary adsorption tests on coarse wool fibers were carried out. First, the coarse wool investigated herein was tested for the adsorption of various cationic and anionic dyes from aqueous solutions (see Figure S3, Supplementary Materials). Results revealed that the coarse wool fibers showed a better performance for the retention of cationic dyes, the adsorption of the cationic dye BB9 being the most promising in terms of both the adsorption capacity and removal efficiency (Figure S3). Second, the influence of the initial pH of the aqueous solution on the efficiency of BB9 adsorption onto wool fibers revealed better performance at naturally occurring levels of pH 6.5 (Figure S4).

**Figure 4:**
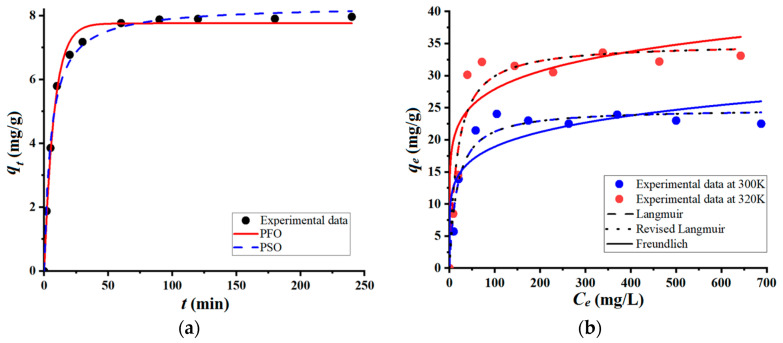
Adsorption (in batch mode) of BB9 cationic dye onto the surface of coarse wool fibers: (**a**) Adsorption kinetics recorded for conditions: *C*_0_ = 50 mg/L, adsorbent dose of 5 g/L, T = 300 K, and pH 6.4 ± 0.2; (**b**) Adsorption isotherms at two distinct temperatures: 300 K and 320 K.

To further assess the effectiveness of coarse wool fibers for removing the cationic organic pollutant (BB9) from wastewater, batch adsorption tests were carried out to reveal the kinetics and isotherms. These adsorption assays enabled us to evaluate the contact time when the equilibrium was reached (from kinetics), as well as the maximum adsorption capacity (from isotherms). The results of adsorption kinetics and isotherms for the retention of BB9 onto the wool adsorbent are shown in Figure 4. As depicted in Figure 4a, the adsorption equilibrium was attained at a contact time equal to 90 min, indicating an adsorption capacity of 7.97 mg/g. Greater contact times (*t* > 90 min) did not significantly change the adsorption capacity, since the stationary state (equilibrium) was achieved. The pseudo-first-order (PFO) and the pseudo-second-order (PSO) kinetic equations were applied to model the experimental data. Likewise, adsorption isotherms were recorded at two values of temperature: 300 K and 320 K (Figure 4b). According to Figure 4b, as the equilibrium concentration (*C_e_*) increases, the adsorption capacity (*q_e_*) increases as well, reaching a stable plateau for a given level of temperature. The observed maximal values of adsorption capacity at equilibrium were 22.50 mg/g at 300 K and 33.09 mg/g at 320 K. It turns out that the equilibrium adsorption capacity (*q_e_*) increased with temperature (Figure 4b). The experimental data regarding the adsorption at equilibrium were interpolated to the Freundlich, Langmuir, and Langmuir-revised isotherm models, using the nonlinear regression analysis. Note that the Langmuir-revised equation, proposed by Azizian and co-workers [16], takes also into account the solubility of the adsorbate (pollutant) in aqueous solutions. For instance, the saturation concentration of BB9 in aqueous solutions is about *C_S_* = 4.36 × 10^4^ mg/L (or 0.1363 mol/L). An advantage of the revised Langmuir model is that it provides a dimensionless constant (*K_ML_*) that can be applied directly for the computation of thermodynamic parameters [16]. The values of parameters of the considered mathematical models (for both kinetics and isotherms) are summarized in Table 1, along with the indicator of the goodness-of-fit (Chi-square test, χ^2^). It should be mentioned that the less the Chi-square value (χ^2^), the better the fit between the experimental data and calculations.

According to the data shown in Figure 4a, and by inspecting chi-square values (*χ*^2^) in Table 1, it may be inferred that the kinetics of adsorption obeys the trend sketched by the PSO equation, rather than by the PFO one. Similarly, by analyzing the isotherms in Figure 4b and the corresponding chi-square values in Table 1, it can be claimed that the Langmuir models better predict the behavior of the adsorption system at equilibrium, when compared to the Freundlich model. It seems that the adsorption of the BB9 dye onto the surface of the wool fiber led to the formation of the molecular monolayer. Regarding the comparison of Langmuir and Langmuir-revised models, both predict similar trends (Figure 4b) with almost identical chi-square tests (Table 1).

In addition, the mean free energy of sorption (also known as average adsorption potential energy) was calculated according to the Dubinin–Radushkevich (D-R) isotherm [20,21]. For the investigated system, the mean free energy of sorption was equal to *E_S_* = 13.66 ± 0.08 kJ/mol, suggesting relevant electrostatic interactions between BB9 cation and the surface of fibers. The D-R isotherm equations [21] and the estimated parameters are also given in Table 1. Likewise, the changes in thermodynamic parameters (i.e., Gibbs free energy, enthalpy, and entropy) were assessed for the studied adsorption process. In this respect, the equilibrium constant was equated to the modified Langmuir constant (*K_ML_*), according to the modern approach as proposed by Azizian et al. [16]. This parameter (*K_ML_*) represents a dimensionless constant, giving the ratio between the elementary rate constants of adsorption (*k_a_*) and desorption (*k_d_*): *K_ML_* = *k_a_*/*k_d_*. For this reason, the modified Langmuir constant (*K_ML_*) can be directly used in thermodynamic computations, without any supplemental mathematical transformations [16,17]. This approach was developed on a solid theoretical background [16], thereby, enabling the ample assessment of the thermodynamic parameters for the adsorption processes from the liquid phase.

As a result of thermodynamics calculations, the following values of the mentioned parameters were established: (a) Δ*G_ad_* = −20.23 ± 0.64 kJ/mol, (b) Δ*H_ad_* = −0.508 kJ/mol, and (c) Δ*S_ad_* = 63.630 J/(mol × K). These results pointed out a spontaneous adsorption process (Δ*G_ad_* < 0), a slight exothermic effect, and the increment of the random collisions at the solid–liquid interface as the temperature increased. The positive entropy change might be attributed to (1) the formation of a less structured or more randomly oriented adsorbed layer on the wool fibers; (2) an increase in the number of possible arrangements of molecules in the adsorbed multilayers; and (3) an increase in the mobility or disorder of water molecules in the vicinity of the adsorption process.

After adsorption, the BB9-loaded fibers represent a spent adsorbent. Several micrographs of the spent adsorbent (loaded wool fibers) are given in Figure S5 (Supplementary Materials) as an example. Additional tests in batch mode were carried out to assess the desorption efficiency of BB9 from the spent adsorbent into different liquid phases or eluents (see Figure S6). Relevant desorption efficiencies of 65.18% and 42.29% were observed in acidic eluents, that is, in 1M solutions of citric acid and hydrochloric acid, respectively (Figure S6). These outcomes suggested that the ion-exchange phenomena could be involved in the adsorption and desorption of the BB9 dye.

The ion exchange mechanism might be attributed to the keratin, which is the major component of wool. Keratin is recognized for its inclusion of carboxyl (–COO^−^) and amino (–NH_3_^+^) groups along the side chains, which play a role in electrostatic interactions, specifically in Coulomb forces [2]. To bring some intrinsic details regarding the mechanism of the interaction between BB9 molecules (cationic form) and a keratin sequence (negatively charged), molecular docking simulations were performed. To this end, the docking computations were performed on a Dell Precision T7910 workstation using the AutoDock-VINA algorithm [22] encompassed in the YASARA Structure v.20.8.23 program [23]. As a receptor, an α-keratin with 93 residues was taken into account in order to simulate a sequence of keratin proteins retrieved from the RCSB Protein Data Bank (RCSB PDB, rcsb.org). The considered keratin sequence, for modeling purposes, contains 16 carboxylic groups –COO^−^ (residues: 15 GLU and 1 ASP) and 12 protonated groups –NH_3_^+^/=NH2^+^ (residues: 5 LYS and 7 ARG) on the side chain, bearing a total net charge of −4. The results of the molecular docking simulation revealed that the interaction between BB9 molecules (ligand) and α-keratin (receptor) took place with a binding affinity of −4.85 kcal/mol and a dissociation constant of 0.28 mM, being stabilized by the hydrophobic contacts. The best pose of the docked complex (BB9/α-keratin) is shown in Figure S7 from Supplementary Materials. In addition, the analysis of the docked complex at the level of Yasara force-field enabled the estimation of the total intermolecular interaction energy, as well as its components, i.e., Van der Waals (VdW) and Coulomb energy interactions (see Table S2, Supplementary Materials). These results disclosed a total interaction energy equal to −62.72 kcal/mol, from which, −26.13 kcal/mol was attributed to the VdW interactions, and −36.69 kcal/mol to Coloumb (electrostatic) interactions (Table S2). These simulation outcomes underlined that the VdW and Coulomb interactions were comparable in magnitude. However, the electrostatic interactions were somewhat greater. Hence, the molecular docking simulation corroborated the contribution of the ion exchange phenomena, which relies on the electrostatic (Coulomb) interactions.

### 3.3. Adsorption in the Fixed-Bed Column: Optimization of Operating Parameters

**Figure 5:**
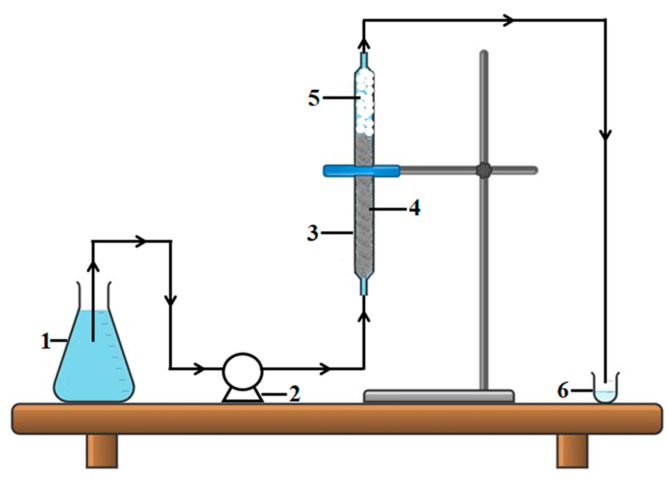
Fixed-bed adsorption set-up (scheme): (1) container with aqueous feed solution (influent); (2) dispensing pump; (3) glass column; (4) fibrous adsorbent material (wool fibers of coarse quality); (5) inert glass beads (for avoiding pressure drop in the column); (6) container for collecting the sample of purified solution (effluent).

Adsorption experiments in the dynamic regime were carried out at room temperature (296 K) by pumping the feed aqueous solutions that were loaded with the BB9 dye (*C*_0_ = 5 mg/L) in the up-flow mode. Thus, the feed solution was pumped through the column (containing wool fibers as the fixed bed) at a controlled flow rate using a dispensing pump, as shown in Figure 5.

The dynamic adsorption studies on the fixed-bed column were carried out by adopting the design of experiments (DoE) and response surface methodology (RSM). Generally, the design of experiments (DoE) allows the efficient sampling of the design space via defining and executing a set of experiments. For this application, a central composite design (CCD) was utilized for experimentations, that is, a face-centered CCD with three center points (Table 2). The effects of two operating parameters (factors) were investigated through the experimental design. Namely, the height (*H*, cm) of the adsorption column and the flow rate of the aqueous solution (*F*_v_, mL/min) were considered as key factors for this application. The output variable (response) tracked in this study was the removal efficiency of the BB9 dye during the dynamic adsorption. This was determined after 240 min of contact between the fibrous adsorbent and the contaminated solution in the column. Hence, for each experimental run in the design of the experiment (Table 2), the process response *Y* (%) was determined experimentally, as given by the following equation:

(5) $$Y=\left(1-\frac{C}{C_{0}}\right)\times 100$$

where *C*_0_ denotes the initial concentration of the BB9 dye (5 mg/L) in the feed aqueous solution (influent), and *C* is the residual concentration of the BB9 dye (pollutant) in the effluent, as determined after a contact time of *t* = 240 min between the adsorbent and pollutant in the dynamic regime. According to the modeling protocol, the values of both operating parameters (*H* and *F*_v_) were scaled to represent these factors as coded variables *x_1_* and *x*_2_, whose values range between −1 (minimum level) and +1 (maximum level). Such a conversion scheme helps to mathematically explore the design space of the factors on the same dimensionless scale (e.g., in a model-based optimization procedure). The mathematical expressions employed for the coding of factors, as well as more details regarding RSM are given elsewhere [24,25]. As summarized in Table 2, the operating parameters are reported as actual factors (*H* and *F*_v_) and as coded factors (*x*_1_ and *x*_2_).

**Figure 6:**
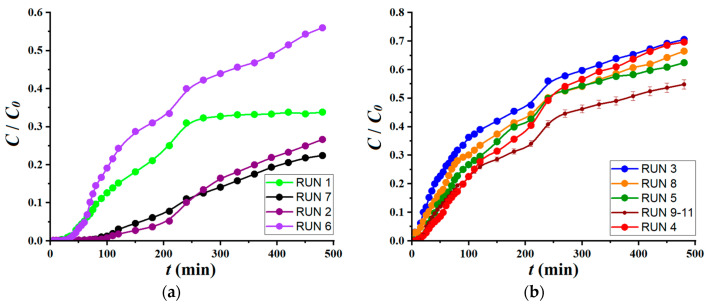
Breakthrough curves determined for different conditions (runs) according to the experimental design: (**a**) kinetics curves with breakthrough points (*t_b_*) greater than 15 min (*t_b_* > 15 min); (**b**) kinetics curves with breakthrough points (*t_b_*) equal or less than 15 min (*t_b_* ≤ 15 min).

The experimental matrix summarized in Table 2 implied 11 experimental runs (trials), where the factors were changed concurrently. Likewise, the response of the dynamic adsorption process (*Y*, %) was determined experimentally for each run (set of conditions). The central assays (runs no. 9 to 11) were carried out to evaluate the reproducibility of the adsorption experiments in the fixed-bed column. It should be mentioned herein that for each condition (run) specified in Table 2, the kinetic profile (relative concentration *C_t_*/*C*_0_ versus time) was determined for a duration of 480 min. All these recorded kinetic profiles (breakthrough curves) are shown in Figure 6. The response (color removal efficiency) observed in the middle of the time span (i.e., 240 min) was used as the output variable in developing the empirical model.

Hence, the mathematical empirical model (second-order polynomial with interaction term) was constructed based on the experimental matrix (Table 2), and by using the multiple regression technique [24,25]. Thus, the estimated response ($\hat{Y}$) can be predicted in terms of coded factors (*x*_1_ and *x*_2_), with the aid of the following data-driven model:

(6) $$\hat{Y}=60.48+6.97x_{1}-15.37x_{2}-3.58x_{1}x_{2}-7.51x_{1}^{2}+12.50x_{2}^{2}$$

subjected to −1 ≤ *x_j_* ≤ +1; (*j* = 1, 2).

The resulting multiple-regression model (Equation (6)) was tested for significance using the analysis of variance (ANOVA) [24]. The statistical descriptors assessed by the ANOVA method are summarized in Supplementary Materials Table S3. The analysis of variance (ANOVA) disclosed a multiple correlation coefficient (*R*^2^) equal to 0.978, suggesting that the constructed model can account for more than 97% of data variation.

**Figure 7:**
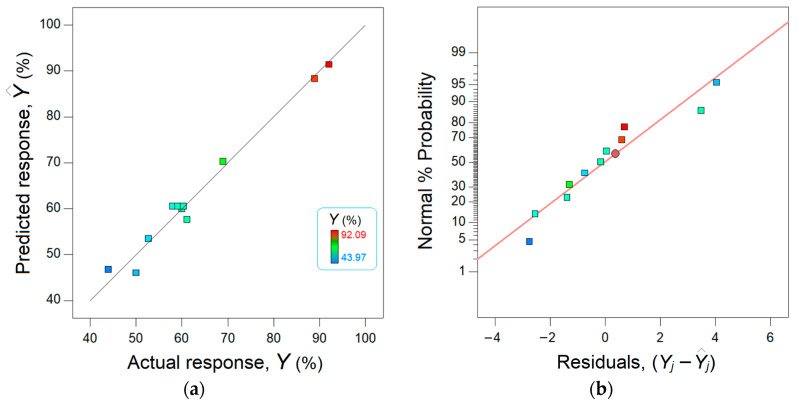
Concordance between actual response (experimental) and predicted response (modeled): (**a**) parity plot and (**b**) normal plot of residuals.

The agreement between the observed data from the experiment and the estimated data from the model is shown in Figure 7. The data points scattered in the vicinity of the bisector (45° straight line) suggest a good concordance between the model and the observations (Figure 7a).

Moreover, Figure 7b shows the normal plot of residuals. Note that the residual error means the difference between the observed response and the response estimated by the model. Thus, this graph (Figure 7b) displays the departure of the residual errors from the normal distribution. As one can see from Figure 7b, the residuals are distributed nearby and on both sights of the straight line, attesting a normal distribution.

The final mathematical model with actual factors (operating parameters) was deduced using the substitution technique. Hence, the empirical model can be expressed in terms of actual factors as follows:

(7) $$\hat{Y}=-188.86+53.02\times H-28.21\times F_{v}-0.89\times H\times F_{v}-1.88{\times H}^{2}+3.12\times F_{v}^{2}$$

subjected to 10.0 ≤ *H* ≤ 14.0 (cm); 3.0 ≤ *F*_v_ ≤ 7.0 (mL/min).

**Figure 8:**
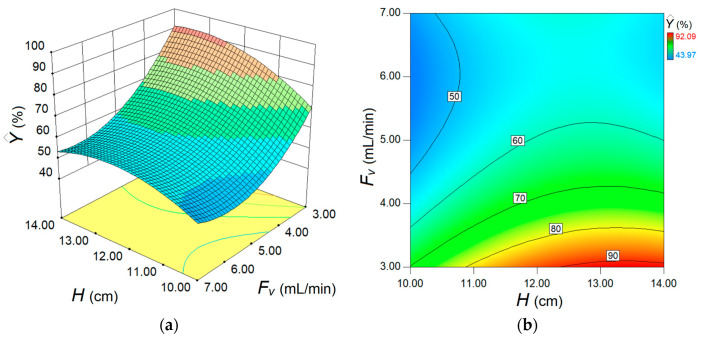
Response surface diagrams depicting the separation performance for fixed-bed adsorption process: (**a**) 3D plot and (**b**) contour-lines 2D map of response surface showing the conjoint effect of fixed bed height *H* (cm) and feed flow rate *F_v_* (mL/min) on the estimated process response.

The final model, relying on actual factors (Equation (7)), was employed to inspect (by simulation) the response surface, providing the 3D and 2D plots (Figure 8). These graphs aim to disclose the synergetic effect of factors on the estimated response.

**Figure 9:**
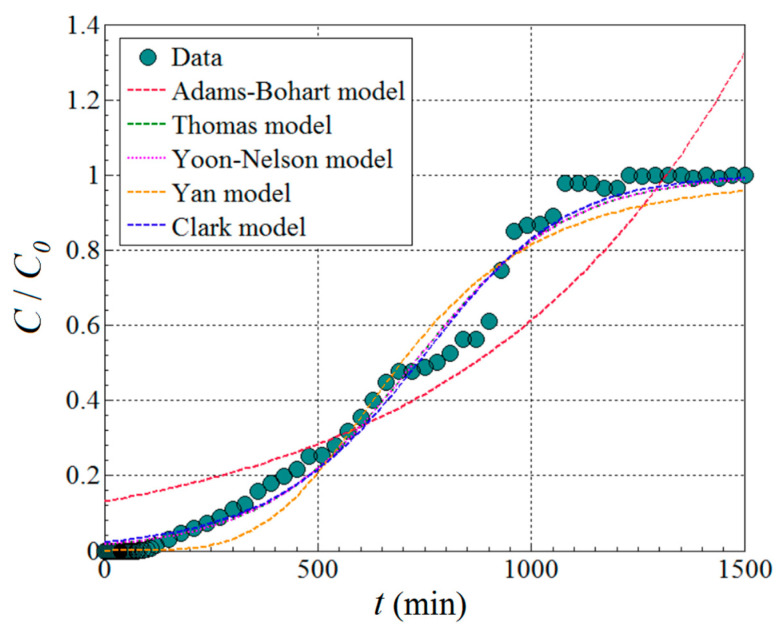
Breakthrough curve recorded under optimal conditions (*H* = 13.5 cm and *F*_v_ = 3.0 mL/min); comparison of theoretical breakthrough curves (dashed lines) with experimental data for the BB9 dye adsorption onto the fixed bed made of wool fibers; *C*_0_ = 5 mg/L and T = 296 K.

As one can see from Figure 8, the main effect of feed flow rate *F_v_* is greater than the main effect of the adsorption bed height *H*, but in the opposite sense. For instance, the increment of the feed flow rate (*F_v_*) in the system depresses the removal efficiency ($\hat{Y}$). In contrast, increasing the height of adsorption column *H* gradually enhances the removal efficiency. However, owing to the interaction effect between these two factors (*F_v_* and *H*), the influence of the *H* factor is more evident at lower levels of flow rate (*F_v_*). At higher flow rates, the effect of adsorption bed height is not so obvious. According to Figure 8, the optimal region emerges for the following domain of factors *F_v_* (3.0–3.5 mL/min) and *H* (12.0–14.0 cm). To pinpoint the optimal conditions more precisely, numerical optimization (model-based) was carried out. To this end, the numerical optimization was completed using the simplex direct search algorithm [26] encompassed in the Design-Expert software (v.10). The optimal conditions indicated by this procedure were *H* = 13.5 cm (adsorption bed height) and *F_v_* = 3.0 mL/min (feed flow rate). Under these indicated conditions, the computed response variable was equal to $\hat{Y}$ = 92.04% (estimated value), whereas the response confirmed experimentally (observed after a contact time of 240 min) was *Y* = 92.56% (actual value). This difference, equal to 0.52%, represents the residual error between the model and experiment. Note that the value of the actual response (92.56%) in optimal conditions was the greatest when compared to any value reported in DoE (Table 2). For the established best running conditions (*H* = 13.5 cm and *F_v_* = 3.0 mL/min), the full kinetic profile of adsorption in the fixed-bed column (i.e., breakthrough curve) was determined for a longer time (Figure 9). These collected data were further subjected to nonlinear regression analysis in order to establish the parameters of the particular kinetic models that were initially employed to assess the performance of the adsorption column. These models can estimate the kinetic profiles (breakthrough curves) for the fixed-bed adsorption systems. In the case of our application, the breakthrough curve recorded under the optimal conditions was modeled using Adams–Bohart, Thomas, Yoon–Nelson, Yan, and Clark kinetic models [15,27,28,29] in order to estimate the dynamic behavior of the system. Nonlinear regression analysis was performed (in Matlab v.9.9) to compute the main parameters of the models. To assess the accordance between the model predictions and experimental data from the breakthrough curve, the error function (*ε*^2^) was calculated. This error function expresses the goodness-of-fit and represents (*ε*^2^ = Σ(*C*(*t*)_exp_ − *C*(*t*)_calc_)^2^) the sum of squares of residual errors. Typically, the less the error function (*ε*^2^) is, the better the prediction given by the model. The explicit mathematical expressions of the kinetic models describing the optimal breakthrough curve are summarized in Table 3, along with the models’ parameters and the estimated error function (*ε*^2^). The smallest values of the error function were found for the Thomas and Yoon–Nelson models, suggesting these models as the best ones in terms of goodness-of-fit. The predictions provided by other models (Clark and Yan) were also relevant. Instead, for the Adams–Bohart model, the highest value of the error function was determined, yet the predictions might be considered satisfactory for the experimentation region (Figure 9).

The adsorption capacity in the dynamic regime (fixed-bed column) can be estimated from the breakthrough curves when applying the following equation [28,29]:

(8) $$q_{x}=\frac{F_{v}}{1000\times m_{S}}\int_{0}^{t_{x}}\left(C_{0}-C_{t}\right)dt$$

where *q_x_* denotes the adsorption capacity (mg/g) attained at a given time *t_x_* (min), *m_S_* is the mass (g) of the adsorbent fixed bed, and *F*_v_ is the flow rate (mL/min) of the feed solution that flows through the fixed bed. Consequently, the amount of pollutant (*λ_x_*, mg) retained into the fixed bed can be readily estimated (*λ_x_* = *q_x_* × *m_S_*). In Equation (8), the variable *C_t_* can be substituted via the explicit relation given by a breakthrough model (i.e., Adams–Bohart, Thomas, Yoon–Nelson, Yan, or Clark, given in Table 3).

Thus, the solving of Equation (8) can be performed by means of the numerical integration technique (e.g., the Simpson method). In our case, the adsorption capacity in the dynamic regime (*q_x_*, mg/g) was determined with Equation (8) for different moments of time (*t_x_*, min) in the breakthrough curve and applying different dynamic models. These results are summarized in Table S4. The considered moments of time from kinetic profile were the breakthrough point (*t_b_* = 90 min, when *C_b_* = 0.05 × *C*_0_), an intermediate point (*t_i_* = 240 min), the saturation or exhausting point (*t_s_* = 1080 min, when the condition *C_s_* = 0.95 × *C*_0_ is met), and the final point in the breakthrough curve (*t_f_* = 1500 min). All these values are reported in Table S4. For example, on the saturation point when the adsorption column began to be exhausted (*C_s_* = 0.95 × *C*_0_), the adsorption capacity was found to be *q_s_* = 5.51 ± 0.04 mg/g, which corresponds to an amount of pollutant removal of *Λ_s_* = 10.64 ± 0.08 mg. Note that all dynamic models considered (Adams–Bohart, Thomas, Yoon–Nelson, Yan, and Clark) converged to these values. It should be emphasized herein that at the breakthrough point (*t_b_* = 90 min), the observed color removal efficiency was equal to *Y_b_* = 99.77%, and for the intermediate point (*t* = 240 min), it was 92.56%. Up to a contact time of 280 min, the color removal efficiency was found to be still around 90%. Afterward, it started to decrease, for instance, it was 74.86% at *t* = 480 min and 2.26% at the saturation point (*t_s_* = 1080 min). Several photo snapshots of the fixed-bed column, at different contact times, are given in Figure S8 from the Supplementary Materials.

Lastly, a comparative analysis was conducted, aiming to correlate the findings from this study with the outcomes reported in the literature (Table 4).

The wool/dye adsorption systems reported in the literature are summarized in Table 4; most of them referred to batch adsorption experiments. In our study involving coarse wool fibers loaded with BB9 dye, the results of adsorption performance under batch conditions fall somewhere in the middle when compared to findings from other studies listed in Table 4.

Regarding fixed-bed adsorption, fibrous materials, other than wool, had been reported for the retention of dyes, such as amino-modified cotton fibers [37], bagasse treated with tartaric acid [38], and chemically modified kenaf core fibers [39] (see Table 4). For fibrous materials like modified cotton [37], it was observed that the adsorption capacity was less for fixed-bed adsorption when compared to batch adsorption for the same system. In our study, working with coarse wool fibers, we observed a similar behavior. This might be attributed to the less intense stirring in the fixed-bed adsorption when compared to batch mode. Hence, in a column-mode adsorption, the dye molecules might not be distributed over the all-adsorptive sites of the material for the considered contact time [37].

However, the utilization of wool fibers as a fixed-bed column and the analysis of the breakthrough curve are reported, to best of our knowledge, for the first time in this study. Thus, in the dynamic regime of adsorption, a removal efficiency of 92.56% was observed for 4 h of operability of the fixed-bed column (*H* = 13.5 cm) at a feed flow rate of *F*_v_ = 3.0 mL/min. This corresponded to passing a total volume of 0.72 L of contaminated solution ([BB9]_0_ = 5 mg/L) through the column. Relaying on experimental evidence, it might be inferred that a single adsorption column (with wool as a fixed bed) can operate in a feasible regime (ensuring > 90% separation efficiency) for up to 280 min. For greater operating times (up to achieving the saturation point), the column might be included in a system of adsorption columns connected consecutively. It should be noted herein that for industrial applications the adsorbent column is usually replaced when the relative concentration *C_t_*/*C*_0_ is equal to value 0.5, that is, 50% breakthrough is attained [39]. For our application (wool fibers/BB9), the relative concentration of 0.5 (i.e., 50% breakthrough) was achieved after 780 min contact time, operating at the inlet flow rate of 3.0 mL/min. Above this point, the column might be still operational until attaining the value *C_t_*/*C*_0_ = 0.9, which represents the operating limit of the adsorption column. For greater values (*C_t_*/*C*_0_ > 0.9), the column becomes less operational, and for *C_t_*/*C*_0_ > 0.95, it becomes completely exhausted. Ultimately, this study highlighted that coarse wool fibers have a relevant potential as low-cost sorbents for the uptake of cationic dyes from wastewater, in both batch modes and in fixed-bed adsorption systems.

## 4. Conclusions

To summarize, coarse wool fibers with an average fiber diameter of 63 μm revealed a relevant efficiency for the retention of the cationic dye (BB9) from wastewater by adsorption. Adsorption kinetics unveiled a contact time greater than 90 min to attain the adsorption equilibrium between wool fibers (adsorbent) and the BB9 cationic dye (adsorbate). The experimental data more expediently obeyed the pseudo-second-order (PSO) equation for the adsorption kinetics. Adsorption isotherms at two distinct temperatures, 300 K and 320 K, indicated the maximum adsorption capacity (observable) of 22.50 mg/g and 33.09 mg/g, respectively. The adsorption data at equilibrium better matched the Langmuir-type isotherm equations (i.e., best fitting to classic Langmuir and Langmuir-revised model). Thermodynamic parameters were assessed by considering the dimensionless equilibrium constant, derived from the Langmuir-revised model. The investigated adsorption process was a spontaneous one, accompanied by a slight exothermic effect (Δ*H_ad_* = −0.508 kJ/mol). The calculated values of the Gibbs free energy (Δ*G_ad_* = −20.23 ± 0.64 kJ/mol) and of the mean free energy of sorption from D-R isotherm (*E_S_* = 14.01 ± 0.42 kJ/mol) probed the contribution of the electrostatic interactions (ion-exchange) for the retention of BB9 cation onto wool fibers. Acidic eluents (C_6_H_8_O_7_ or HCl) were best suited for the desorption of retained BB9 from the spent wool.

The dynamic adsorption of the BB9 cationic dye onto the fixed-bed column made of wool fibers was modeled and optimized by means of the response surface methodology, aiming to maximize the separation efficiency. The model-based numerical optimization indicated the following recommended values of operating parameters: *H* = 13.5 cm (adsorption bed height made of wool fibers) and *F*_v_ = 3.0 mL/min (flow rate of feed aqueous solution). Hence, the fixed-bed adsorption experiments were better performed when the system operated at a reduced dye concentration in the influent, higher bed height of the adsorbent column, and a lower feed flow rate. The low value of the feed flow rate may be viewed as a drawback for treating large volumes of water. By contrast, the same low feed flow rate ensures a better contact (adsorbent-adsorbate) and consequently a better water purification, which is an advantage from the separation standpoint. Under these optimal conditions, the observed pollutant removal efficiency was found to be 99.77% at a contact time of 90 min (breakthrough point), and 92.56% for a contact time equal to 240 min. Moreover, the adsorption column (with wool as a fixed bed) can operate in a feasible regime of *H* = 13.5 cm and *F*_v_ = 3.0 mL/min (ensuring > 90% separation efficiency) for a contact time up to 280 min. The data from the breakthrough curve recorded under the optimal conditions were best interpolated by the kinetic models of Thomas and Yoon–Nelson. At the point of saturation, where the adsorption column commenced depletion (*C_S_* = 0.95 × *C*_0_), the adsorption capacity was determined to be *q_s_* = 5.51 ± 0.04 mg/g using numerical integration, corresponding to a pollutant removal amount of *λ_s_* = 10.64 ± 0.08 mg. Ultimately, it can be asserted that coarse wool fibers represent a low-cost product that proved to be an efficient adsorbent for the retention of cationic organic pollutant, in both batch and dynamic regimes.

## Figures and Tables

**Table 1:** Mathematical models and estimated parameters describing adsorption kinetics and isotherms for BB9 retention onto coarse wool fibers.

Model	Type	Model Equation	Model Parameters
PFO	Kinetics (at T = 300 K)	$q_{t}=q_{e}\left(1-e^{-k_{1}t}\right)$	*q_e_* = 7.759 (mg/g)
			*k_1_* = 1.306 × 10^−1^ (min^−1^)
			*χ*^2^ = 0.056
PSO	Kinetics (at T = 300 K)	$q_{t}=\frac{k_{2}q_{e}^{2}t}{1+k_{2}q_{e}t}$	*q_e_* = 8.321 (mg/g) *k_2_* = 2.268 × 10^−2^ (min^−1^) *χ*^2^ = 0.050
Freundlich	Isotherm (at T = 300 K)	$q_{e}=K_{F}C_{e}^{1/n_{F}}$	*K_F_* = 8.855 (mg/g)(L/mg)^1/*n*^*_F_* *n_F_* = 6.067 *χ*^2^ = 14.120
	Isotherm (at T = 320 K)		*K_F_* = 14.782 (mg/g)(L/mg)^1/*n*^*_F_* *n_F_* = 7.256 *χ*^2^ = 3.837
Langmuir	Isotherm (at T = 300 K)	$q_{e}=\frac{q_{m}K_{L}C_{e}}{1+K_{L}C_{e}}$	*q_m_* = 24.855 (mg/g) *K_L_* = 5.926 × 10^−2^ (L/mg) *χ*^2^ = 3.310
	Isotherm (at T = 320 K)		*q_m_* = 35.036 (mg/g) *K_L_* = 5.845 × 10^−2^ (L/mg) *χ*^2^ = 9.886
Langmuir Revised	Isotherm (at T = 300 K)	$q_{e}=\frac{q_{ML}K_{ML}C_{e}}{\left(C_{S}-C_{e}\right)+K_{ML}C_{e}}$	*q_ML_* = 24.847 (mg/g) *K_ML_* = 2.584 × 10^+3^ *χ*^2^ = 3.310
	Isotherm (at T = 320 K)		*q_ML_* = 35.023 (mg/g) *K_ML_* = 2.549 × 10^+3^ *χ*^2^ = 9.886
Dubinin–Radushkevich ($\varepsilon_{P}$ -Polanyi potential)	Isotherm (at T = 300 K)	$q_{e}=q_{D}exp\left(-K_{D}\varepsilon_{P}\right)$ $\varepsilon_{P}=R_{g}Tln\left(1+\frac{1}{C_{e}}\right)$ $E_{s}=\frac{1}{\sqrt{2K_{D}}}$	*q_D_* = 0.05323 (g/g) *K_D_* = 2.656 × 10^−3^ (mol^2^/kJ^2^) *E_S_* = 13.72 (kJ/mol) *χ*^2^ = 7.643 × 10^−3^
	Isotherm (at T = 320 K)		*q_D_* = 0.07689 (g/g) *K_D_* = 2.698 × 10^−3^ (mol^2^/kJ^2^) *E_S_* = 13.61 (kJ/mol) *χ*^2^ = 1.216 × 10^−2^

**Table 2:** DoE: Central composite design (face-centered) used for fixed-bed adsorption (WF/BB9).

Run	Column Height (cm)		Feed Flow Rate (mL/min)		Color Removal Efficiency: Response Determined after 240 min Contact Time
	Coded	Actual	Coded	Actual	
	*x*_1_	*H*, cm	*x*_2_	*F*_v_, mL/min	Y (%)
1	−1	10.0	−1	3.0	68.99
2	+1	14.0	−1	3.0	92.09
3	−1	10.0	+1	7.0	43.97
4	+1	14.0	+1	7.0	52.76
5	−1	10.0	0	5.0	50.05
6	+1	14.0	0	5.0	59.98
7	0	12.0	−1	3.0	88.95
8	0	12.0	+1	7.0	61.11
9	0	12.0	0	5.0	57.94
10	0	12.0	0	5.0	59.10
11	0	12.0	0	5.0	60.32

**Table 3:** Kinetics models and parameters for the dynamic adsorption of BB9 onto wool fibers placed as fixed bed in column; *C*_0_ = 5 mg/L and T = 296 K.

Model	Model Equation	Model Parameters
Adams–Bohart	$\frac{C_{t}}{C_{0}}=exp\left(k_{A}C_{0}t-k_{A}N_{0}\frac{H}{u}\right)$	*k_A_* = 3.0858 × 10^−4^ (L mg^−1^ min^−1^) *N*_0_ = 974.93 (mg/L) [*H* = 13.5 (cm)] ^(a)^ [*u* = 2.0 (cm/min)] *ε*^2^ = 1.3979
Thomas	$\frac{C_{t}}{C_{0}}=\frac{1}{1+exp\left(k_{T}q_{T}\frac{m_{S}}{F_{L}}-k_{T}C_{0}t\right)}$	*k_T_* = 1.1215 × 10^−3^ (L mg^−1^ min^−1^) *q_T_* = 5.6326 (mg/g) [*m_S_* = 1.930 (g)] [*F_L_* = 3.0 × 10^−3^ (L/min)] *ε*^2^ = 0.1238
Yoon–Nelson	$\frac{C_{t}}{C_{0}}=\frac{exp\left(k_{Y}t-k_{Y}\tau \right)}{1+exp\left(k_{Y}t-k_{Y}\tau \right)}$	*k_n_* = 5.6074 × 10^−3^ (min^−1^) *τ* = 724.72 (min) *ε*^2^ = 0.1239
Yan	$\frac{C_{t}}{C_{0}}=1-\frac{1}{1+{\left(\frac{C_{0}F_{L}t}{q_{Y}m_{S}}\right)}^{a_{Y}}}$	*a_Y_* = 4.0632 *q_Y_* = 5.4017 (mg/g) [*m_S_* = 1.930 (g)] [*F_L_* = 3.0 × 10^−3^ (L/min)] *ε*^2^ = 0.2512
Clark	$\frac{C_{t}}{C_{0}}={\left(\frac{1}{A_{C}\times \exp\left(-r_{C}t\right)+1}\right)}^{\frac{1}{n_{F}-1}}$	*A_C_ =* 6.6702 × 10^6^ *r_C_* = 1.5722 × 10^−2^ (min^−1^) [*n_F_* = 6.067 (Freundlich constant)] *ε*^2^ = 0.13361

^(a)^ parameters in square brackets [*H*, *u*, *m_S_*, *F_L_*, and *n_F_*] were fixed at their actual values.

**Table 4:** Comparison of different fibrous adsorbents for the retention of dyes from liquid phases.

Adsorbent	Dye ^1^	Adsorption Capacity (mg/g)	Removal Efficiency (%)	Operation Mode	Reference
Wool fibers (WF)	ARS	91.5	93.2	Batch	[11]
Wool fibers (WF)	CR	3.9	-	Batch	[12]
Irradiated WF	CR	3.3	-	Batch	[12]
Wool powder	MB	107.5	30.0	Batch	[13]
Wool fibers (WF)	AcR1	4.0	-	Batch	[14]
Carbonized wool	BR22	68.8	5.5	Batch	[30]
Modified wool	Rh6G	282.6	-	Batch	[31]
Wool/PVA	MB	238.7	50.0	Batch	[32]
Wool fibers (WF)	NR4	86.9	-	Batch	[33]
Woolen yarn	*A. vasica*	7.2	-	Batch	[34]
Hydrolysed WF	RhB	294.0	90.0	Batch	[35]
Wool waste	AcR337	15.7	42.0	Batch	[36]
m-Cotton fibers ^2^	AB25	618.0	40.45	Fixed-Bed	[37]
m-Bagasse	MB	0.13	94.22	Fixed-Bed	[38]
m-Kenaf fibers	AB25	162.7	-	Fixed-Bed	[39]
Coarse WF ^3^	BB9 (MB)	24.8	86.6	Batch	This study
Coarse WF ^4^	BB9 (MB)	5.6	92.56	Fixed-Bed	This study

^1^ ARS (Alizarin red S); CR (Congo Red); AcR1 (Acid Red 1); BR22 (Basic Red 22); Rh6G (Rhodamine 6G); NR4 (Natural Red 4); *A. vasica* (*Adhatoda vasica* natural dye); RhB (Rhodamine); AcR337 (Acid Red 337); AB25 (Acid Blue 25); BB9 (Basic Blue 9) synonym to MB (Methylene Blue); ^2^ Prefix m- stands for modified (chemically); ^3^ [BB9]_0_ = 50 mg/L and 0.25 g adsorbent; ^4^ [BB9]_0_ = 5 mg/L, 1.93 g adsorbent, and 3.0 mL/min feed flow rate.

## Data Availability

Data is contained within the article or Supplementary Materials.

## Supplementary Materials

The following supporting information can be downloaded at: https://www.mdpi.com/article/10.3390/ma17040790/s1, Figure S1: Mechanical properties of wool: Stress–strain curves for dry and wet wool fibers; Table S1: Mechanical properties of wool fiber samples; Figure S2: Infrared spectrum (FTIR) for the investigated coarse wool fiber; Figure S3: Comparative analysis of the performance of coarse wool fibers for adsorption of various dyes dissolved in aqueous solutions, i.e., cationic dyes (BB9, BG, Rh-6G) and anionic dyes (AR27, CR, EB); dyes’ abbreviations: BB9—basic blue 9; BG—brilliant green; Rh-6G—rhodamine 6G; AR27—acid red 27; CR—congo red; EB—Evans blue; experimental conditions: initial dye concentration of 50 mg/L, adsorbent dose 0.5% *w*/*v*, T = 300 K and 2 h contact time; Figure S4: Effect of initial pH of aqueous solutions on the adsorption BB9 cation dye onto the surface of coarse wool fibers; experimental conditions: *C*_0_ = 50 mg/L, adsorbent dose 0.5% *w*/*v*, *T* = 300 K, and contact time of 2 h; Figure S5: Micrographs from optical microscopy with polarized light showing: (a) pristine (non-loaded) coarse wool fibers; (b, c, d) spent coarse wool fibers loaded with BB9 cationic dye; Figure S6: Desorption efficiency of BB9 dye from spent adsorbent (loaded wool fibers) in different eluent solutions (desorption time 2 h and working volume 15 mL); Figure S7: Molecular docking simulation outcomes: (a) best docking pose of MB (ligand molecule) onto the α-keratin (receptor macromolecule taken from PDB ID: 6JFV) and (b) zoom-in image of the binding site highlighting the hydrophobic contacts; Table S2: Energy of intermolecular interactions between BB9 (ligand) and α-Keratin (receptor) computed by molecular mechanics theory at the level of Yasara force field; Table S3: ANOVA assay for the built mathematical model *Ŷ*(*x_1_*, *x_2_*); Table S4: Adsorption capacity and retained amount of pollutant calculated at different times from breakthrough kinetic models (*m_S_* = 1.93 g, *F*_v_ = 3 mL/min, *H* = 13.5 cm, *C*_0_ = 5 mg/L); Figure S8: Photo-images (snapshots) showing the degree of BB9 loading onto wool fibers in the fixed bed column at different time intervals: (a) 0 min; (b) 120 min; (c) 210 min; (d) 480 min; (e) 780 min and (f) 1500 min.
