# Supplementary material for: Dynamic Adsorption of a Cationic Dye onto Wool Fibers as Column-Filling Media: Response Surface Optimization and Fixed-Bed Adsorption Modeling
Source: Materials (Basel). 2024 Feb 6;17(4):790. doi: 10.3390/ma17040790 (PMC10890706; doi:10.3390/ma17040790)
Supplement: Supplementary file 1 [file materials-17-00790-s001.zip › materials-2840909-supplementary.pdf]

Supplementary Materials

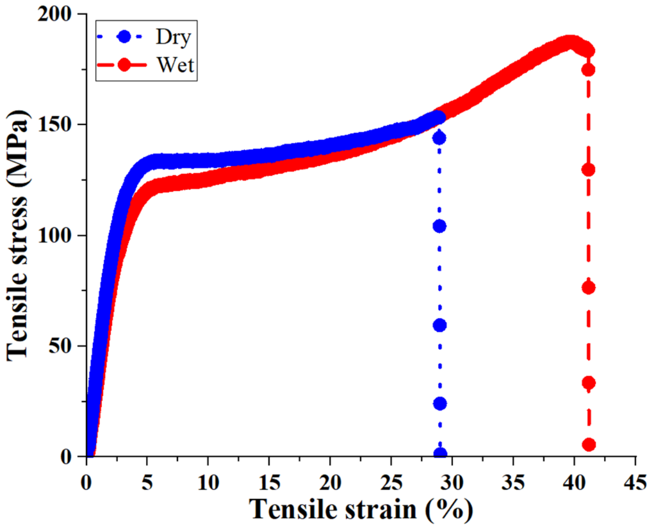

Figure S1. Mechanical properties of wool: Stress-strain curves for dry and wet wool fibers.

Table S1. Mechanical properties of wool fiber samples.

Sample	Tensile strain (%) (Elongation at break)	Tensile stress (MPa)	Young's modulus (GPa)
Dry Wool Fiber (RH 0%)	28.7	153.5	4.72
Wet Wool Fiber (RH 55%)	39.9	187.5	4.23

Note: RH – relative humidity

Comments on **Figures S1** and **Table S1***:* The investigated samples were analyzed in accordance with ASTM standard D 412-06a Standard Test Methods for Vulcanized Rubber and Thermoplastic Elastomers - Adjusted Stress. **Figure S1** shows typical tensile stress-strain curves for dry and wet wool fiber. The stress-strain curves of wool fibers were measured under traction actions at 23 °C and in the conditions of distinct levels of relative humidity (RH); that is 0 % (dry fiber) and RH 55% (wet fiber). The mechanical parameters, such as elongation at break (tensile strain), tensile stress or strength at break, and modulus of elasticity (Young's modulus), were determined, and these values are summarized in **Table S1**. It was observed that tensile strain and stress were greater for wet fiber compared to the dry one. Young's modulus (calculated for linear elastic region) did not change too much for dry and wet wool, describing their ability to resist elastic deformation under load. Overall, the percentage of moisture affects the stress-strain diagram of wool fibers. The higher the RH, the more the fiber can be stretched. Experimental data showed that the dry fiber broke at about 29% elongation, while a wet fiber broke at about 40% elongation. In dynamic adsorption, fibers experience continuous flow and mechanical agitation. Mechanical testing can help assess the fibers' resistance to wear and tear under dynamic conditions. This is important for long-term performance and sustainability of the adsorption process. Likewise, mechanical testing outcomes are useful in assessing the fibers' ability to withstand multiple adsorption-desorption cycles (i.e., their potential for repeated use).

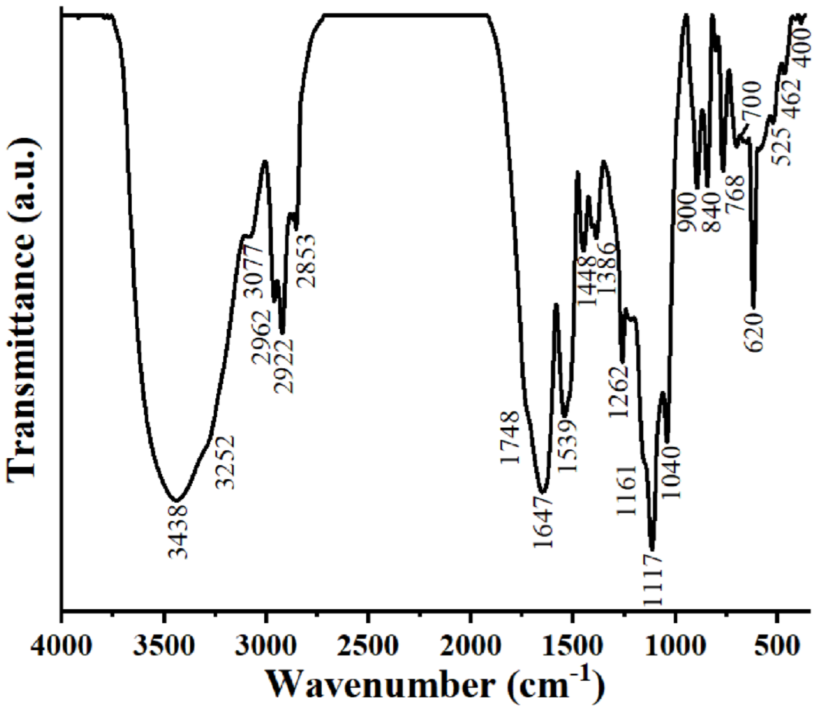

Figure S2. Infrared spectrum (FTIR) for the investigated coarse wool fiber.

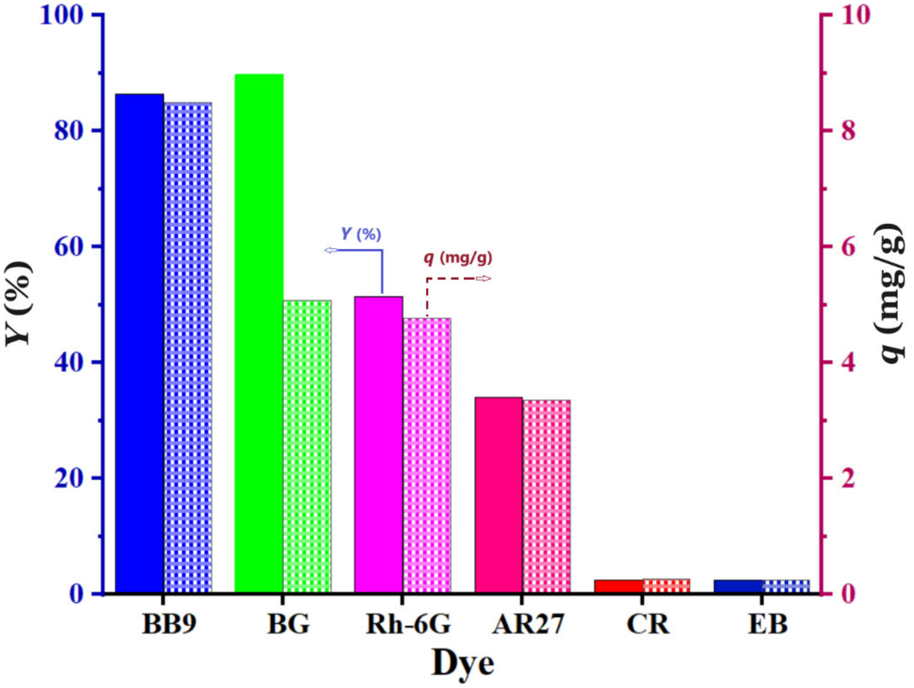

Figure S3. Comparative analysis of the performance of coarse wool fibers for adsorption of various dyes dissolved in aqueous solutions, i.e., cationic dyes (BB9, BG, Rh-6G) and anionic dyes (AR27, CR, EB); dyes' abbreviations: BB9 - basic blue 9; BG - brilliant green; Rh-6G - rhodamine 6G; AR27 - acid red 27; CR - congo red; EB - Evans blue; experimental conditions: initial dye concentration of 50 mg/L, adsorbent dose 0.5% w/v, T=300K and 2h contact time.

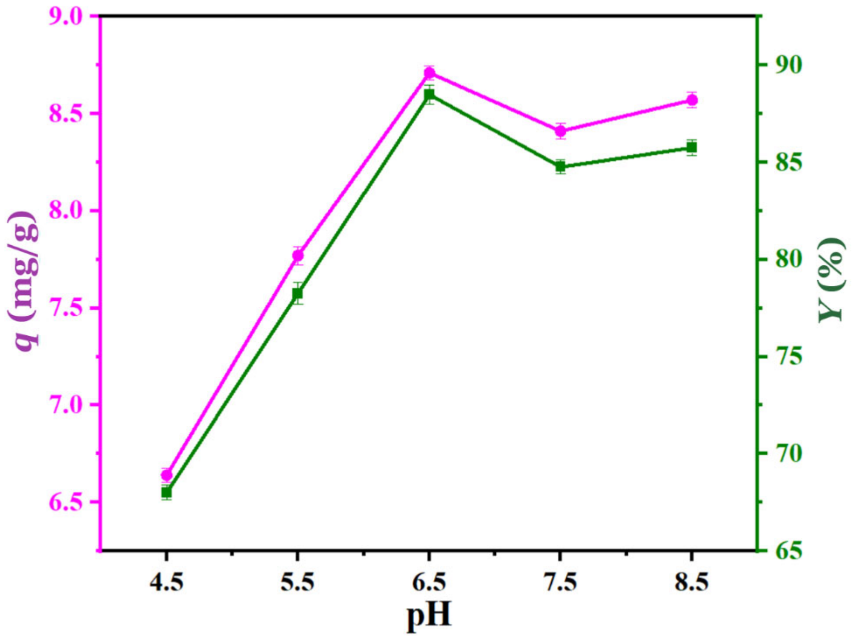

Figure S4. Effect of initial pH of aqueous solutions on the adsorption BB9 cation dye onto the surface of coarse wool fibers; experimental conditions: C_0=50 mg/L, adsorbent dose 0.5% w/v, T=300K, and contact time of 2 h.

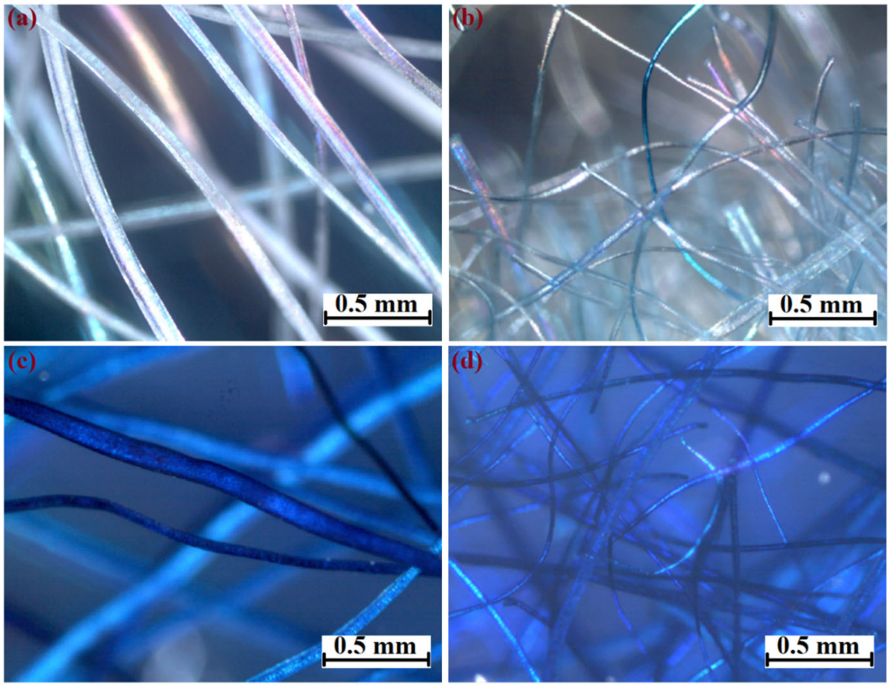

Figure S5. Micrographs from optical microscopy with polarized light showing: (a) pristine (non-loaded) coarse wool fibers; (b, c, d) spent coarse wool fibers loaded with BB9 cationic dye.

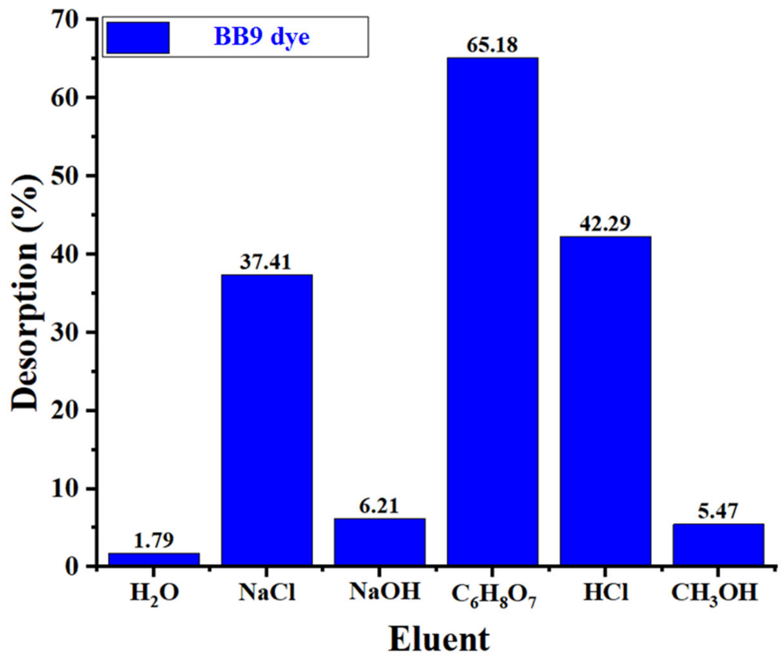

Figure S6. Desorption efficiency of BB9 dye from spent adsorbent (loaded wool fibers) in different eluent solutions of 1M concentration (desorption time 2 h and working volume 15 mL).

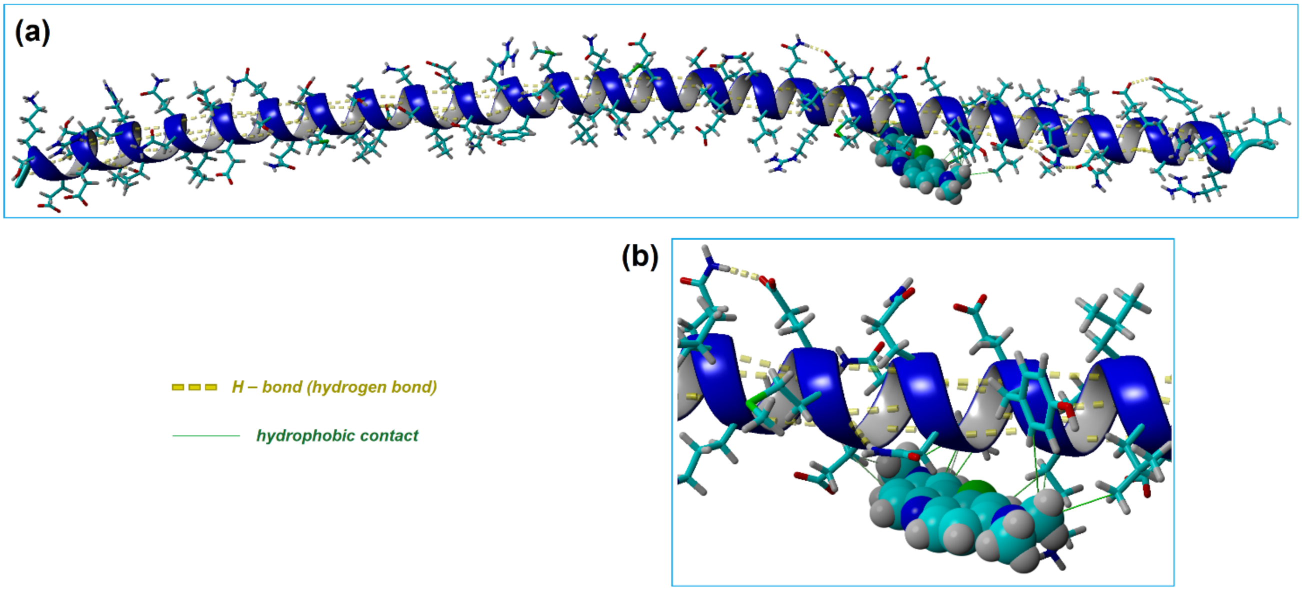

Figure S7. Molecular docking simulation outcomes: (a) best docking pose of MB (ligand molecule) onto the α-keratin (receptor macromolecule taken from PDB ID: 6JFV) and (b) zoom-in image of the binding site highlighting the hydrophobic contacts.

Table S2. Energy of intermolecular interactions between BB9 (ligand) and α-Keratin (receptor) computed by molecular mechanics theory at the level of Yasara force field.

Docking system	Total energy of intermolecular interaction, $\Delta E^{(\text{Total})}$, kcal/mol	VdW intermolecular interaction energy, $\Delta E^{(\text{VdW})}$, kcal/mol	Coulomb intermolecular interaction energy, $\Delta E^{(\text{Coulomb})}$, kcal/mol
BB9 / α-Keratin	–62.72	–26.13	–36.59

Note: *Mathematical relationships for interaction energies calculations, where E is potential energy according to the force field:*

$$\Delta E^{(\text{Total})} = E^{(\text{Total})}_{\text{Complex}} - \left(E^{(\text{Total})}_{\text{Receptor}} + E^{(\text{Total})}_{\text{Ligand}} \right) \qquad \Delta E^{(\text{Total})} \cong \Delta E^{(\text{VdW})} + \Delta E^{(\text{Coulomb})}$$

$$\Delta E^{(\text{VdW})} = E^{(\text{VdW})}_{\text{Complex}} - \left(E^{(\text{VdW})}_{\text{Receptor}} + E^{(\text{VdW})}_{\text{Ligand}} \right) \qquad \Delta E^{(\text{Coulomb})} = E^{(\text{Coulomb})}_{\text{Complex}} - \left(E^{(\text{Coulomb})}_{\text{Receptor}} + E^{(\text{Coulomb})}_{\text{Ligand}} \right)$$

Table S3. ANOVA assay for the built mathematical model $\hat{Y}(x_1, x_2)$.

Source	DF [(a)]	SS [(b)]	MS [(c)]	*F-value* [(d)]	*p-value* [(e)]	R^2 [(f)]	R_{adj}^2 [(g)]
Model	2202.82	5	440.56	46.27	0.0003	0.978	0.957
Residual	47.62	5	9.52				
Total	2250.44	10					

[(a)] degree of freedom; [(b)] sum of squares; [(c)] mean square; [(d)] ratio between mean squares; [(e)] probability of randomness (noise); [(f)] coefficient of multiple correlation; [(g)] adjusted coefficient of determination.

Comments on **Table S3**: Outcomes of ANOVA (**Table S3**) indicate an *F*-value equal to 46.27 and a quite small *p*-value (0.0003) suggesting a significant model from a statistical viewpoint. That is, this model was validated and can be employed to make predictions regarding the process performance in the designed space (region of experimentation). Furthermore, the value of the multiple-correlation coefficient R^2 indicates that the empirical model can explain more than 97% of data variation. Likewise, the adjusted coefficient R^2_{adj} is quite close to R^2, disclosing that the data-driven model containing main, interaction, and quadratic effects provides reliable predictions.

Table S4. Adsorption capacity and retained amount of pollutant calculated at different times from breakthrough kinetic models (m_S=1.93 g, F_v=3 mL/min, H=13.5 cm, C_0=5 mg/L).

**Model**	Adsorption capacity (q_x) [mg / g] in dynamic regime	Retained amount ($\Lambda_x = q_x \times m_S$) of pollutant [mg] on fixed bed
Adams-Bohart	q_b = 0.601	Λ_b =1.160
	q_i = 1.569	Λ_i = 3.028
	q_s = 5.556	Λ_s =10.723
	q_f = 5.630	Λ_f =10.865
Thomas	q_b = 0.684	Λ_b =1.321
	q_i =1.800	Λ_i = 3.475
	q_s = 5.479	Λ_s =10.574
	q_f = 5.638	Λ_f =10.882
Yoon-Nelson	q_b = 0.684	Λ_b =1.320
	q_i = 1.800	Λ_i = 3.475
	q_s = 5.479	Λ_s =10.574
	q_f = 5.638	Λ_f =10.882
Yan	q_b = 0.699	Λ_b =1.350
	q_i = 1.860	Λ_i = 3.591
	q_s = 5.553	Λ_s =10.716
	q_f = 5.816	Λ_f =11.225
Clark	q_b = 0.663	Λ_b =1.280
	q_i = 1.741	Λ_i = 3.359
	q_s = 5.491	Λ_s =10.597
	q_f = 5.516	Λ_f =10.646

Note on subscripts:
b – break point (when C_b is 5% from C_0, or $C_b = 0.05 \times C_0$), t_b = 90 min → Y_b = 99.77% (observed value);
i – intermediary point in breakthrough curve, t_i = 240 min → Y_i = 92.56% (observed value);
s – saturation point (when C_s is 95% from C_0, or $C_b = 0.95 \times C_0$), t_s = 1080 min → Y_s = 2.26% (obs.);
f – final point in breakthrough curve, t_f = 1500 min → Y_f = 0.20% (observed value).

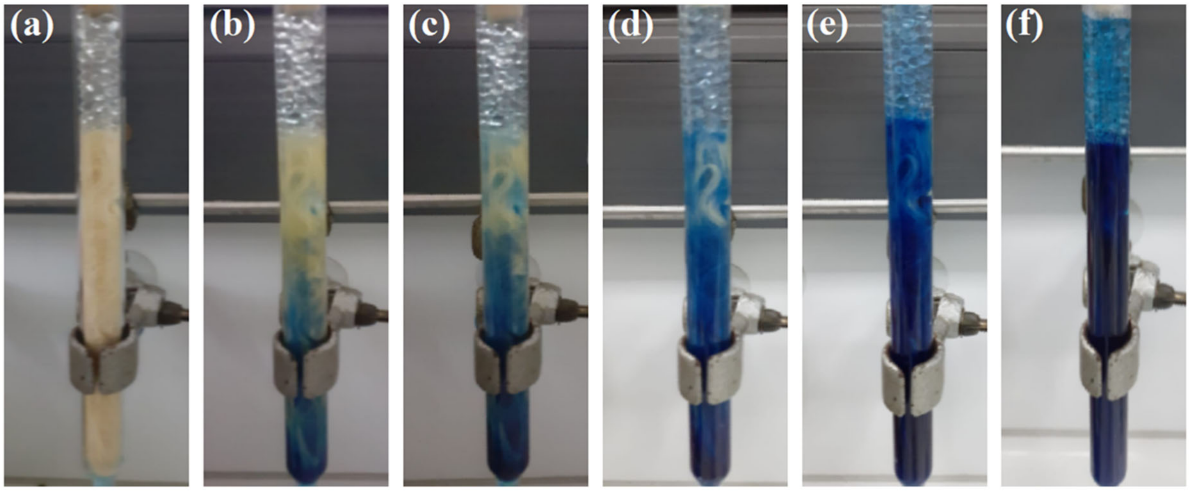

Figure S8. Photo-images (snapshots) showing the degree of BB9 loading onto wool fibers in the fixed bed column at different time intervals: (a) 0 min; (b) 120 min; (c) 210 min; (d) 480 min; (e) 780 min and (f) 1500 min.
